# Comparisons in temperature and photoperiodic-dependent diapause induction between domestic and wild mulberry silkworms

**DOI:** 10.1038/s41598-021-87590-4

**Published:** 2021-04-13

**Authors:** Takeshi Yokoyama, Shigeru Saito, Misato Shimoda, Masakazu Kobayashi, Yoko Takasu, Hideki Sezutsu, Yoshiomi Kato, Makoto Tominaga, Akira Mizoguchi, Kunihiro Shiomi

**Affiliations:** 1 Department of United Graduate School of Agricultural Science, Tokyo University of Agriculture and Technology, Fuchu, 183-8509 Japan; 2 Division of Cell Signaling, National Institute for Physiological Sciences, National Institutes of Natural Sciences, Okazaki, Japan; 3 Gunma Sericultural Technology Center, Maebashi, 371-0852 Japan; 4 Faculty of Textile Science and Technology, Shinshu University, Ueda, Nagano 386–8567 Japan; 5 National Institute of Agrobiological Sciences (NIAS), Tsukuba, 305-8602 Japan; 6 International Christian University, Mitaka, 181-8585 Japan; 7 Division of Liberal Arts and Sciences, Aichi Gakuin University, Nisshin, 470-0195 Japan

**Keywords:** Developmental biology, Entomology

## Abstract

The bivoltine strain of the domestic silkworm, *Bombyx mori,* has two generations per year. It shows a facultative diapause phenotype determined by environmental conditions, including photoperiod and temperature, and nutrient conditions during embryonic and larval development of the mother. However, it remains unclear how the environmental signals received during development are selectively utilized as cues to determine alternative diapause phenotypes. We performed a comparative analysis between the Kosetsu strain of *B. mori* and a Japanese population of the wild mulberry silkworm *B. mandarina* concerning the hierarchical molecular mechanisms in diapause induction. Our results showed that for the Kosetsu, temperature signals during the mother’s embryonic development predominantly affected diapause determination through the thermosensitive transient receptor potential ankyrin 1 (TRPA1) and diapause hormone (DH) signaling pathways. However, embryonic diapause in *B. mandarina* was photoperiod-dependent, although the DH signaling pathway and thermal sensitivity of TRPA1 were conserved within both species. Based on these findings, we hypothesize that TRPA1-activated signals are strongly linked to the signaling pathway participating in diapause induction in Kosetsu to selectively utilize the temperature information as the cue because temperature-dependent induction was replaced by photoperiodic induction in the TRPA1 knockout mutant.

## Introduction

In the domestic silkworm *Bombyx mori*, progeny diapause is induced during the early embryonic stage. The development of diapause-destined embryos is arrested during the G2 cell cycle stage immediately after forming the cephalic lobe and telson and sequential segmentation of the mesoderm and ectoderm^1^. In bivoltine strains, populations of *B. mori* that have two generations per year show a facultative diapause phenotype, which is determined by environmental conditions, such as photoperiod, temperature, and nutrient conditions during embryonic and larval development in the mother’s generation^2^. In general, progeny diapause of bivoltine strains is predominantly determined by environmental conditions during maternal embryonic development in conjunction with long days and high temperatures that induce diapause eggs^2–4^. However, the environmental cues that induce diapause are different in each bivoltine strain and are also updated by integrating cumulative signals received through development^5,6^. For instance, when larvae were reared on an artificial diet under long-day conditions, some silkworm strains produced non-diapause eggs even if the eggs were incubated in diapause egg-producing conditions^2,7^. However, it remains unclear how environmental signals received through development are selectively utilized as cues to determine alternative diapause phenotypes.

For the bivoltine strain Kosetsu, the temperature signal during the mother’s embryonic development is the predominant factor affecting diapause determination, regardless of the photoperiods during embryonic and larval development under our rearing condition using an artificial diet^8,9^. When incubating eggs at 25 °C under continuous darkness (25DD), the resultant female moths lay nearly 100% diapause eggs. In contrast, incubation of eggs at 15 °C in dark conditions (15DD) resulted in moths that lay nearly 100% non-diapause eggs. Thus, Kosetsu is considered a typical strain that is susceptible to temperature-dependent diapause induction.

The molecular mechanisms underlying the induction of *B. mori* embryonic diapause have been investigated extensively^5,9^. Our previous study revealed that embryonic diapause is induced by the diapause hormone (DH) signaling pathway, consisting of a highly sensitive and specific interaction between DH and DH receptors (DHR) pupal-adult development^8^. *DH-PBAN* encodes a polyprotein precursor containing DH, pheromone biosynthesis activating neuropeptide (PBAN), and α-, β-, and γ-subesophageal ganglion (SG) neuropeptides (SGNPs). DH is produced from the DH-PBAN precursor through posttranslational processing^10^. It is produced exclusively in seven pairs of neurosecretory cells, known as DH-PBAN-producing neurosecretory cells (DHPCs), located within the SG in the mother’s generation^11^. DH is released from the corpus cardiacum (CC)^12^, a major release site for neuropeptide hormones, into the hemolymph, and acts on DHR in ovary^13^. Furthermore, the cerebral γ-aminobutyric acid (GABA)ergic and corazonin (Crz) pathway modulates DH release throughout the temperature-dependent expression of the plasma membrane GABA transporter^9^. Recent studies revealed that the transient receptor potential ankyrin 1 (TRPA1) (BmoTRPA1) acts as a thermosensitive channel activated at temperatures above ~ 21 °C and affects diapause induction through DH release in Kosetsu. Thus, BmoTRPA1 acts as a molecular switch for the temperature-dependent diapause induction in Kosetsu^14^.

*B. mori* was domesticated from the wild mulberry silkworm, *B. mandarina*, approximately 5000–10,000 years ago in China^15–18^. *B. mori* and *B. mandarina* are similar in morphological, cytological, and physiological aspects; copulation between them is possible, and the resultant progeny is never sterile^19–21^, although both species are clearly genetically differentiated^22^. In addition, *B. mandarina* enters embryonic diapause at a similar developmental stage as in *B. mori*^23,24^. The bi-, tri-, and univoltine *B. mandarina* inhabit Japan based on the moth’s seasonal fluctuation^25,26^. Furthermore, photoperiodic-dependent diapause induction of *B. mandarina* was shown in a previous report, in which more female moths produced diapause eggs under short-day conditions, such as a 12-h light/12-h dark cycle (12L12D) than under long-day conditions, such as 15L9D during larval stages, even if the temperature was kept constant at 25 °C during embryonic development. Diapause induction is most strongly affected by photoperiod in a certain developmental stage following the second larval ecdysis^27^. Thus, to our knowledge, *B. mandarina* enters diapause by photoperiodic induction, which contrasts with the temperature-dependent diapause induction in Kosetsu of *B. mori*. Therefore, we performed a comparative analysis of diapause induction using Kosetsu and a Japanese population of *B. mandarina* with respect to the hierarchical molecular mechanisms to elucidate the selectivity and integration of environmental cues. In the present study, we hypothesize that TRPA1-mediated signals are strongly linked to the signaling pathway participating in diapause induction in Kosetsu to selectively utilize the temperature information as the cue.

## Results and discussion

### Temperature and photoperiodic-dependent diapause induction are different between the Kosetsu strain of *B. mori* and the Fuchu population of *B. mandarina*

**Figure 1:**
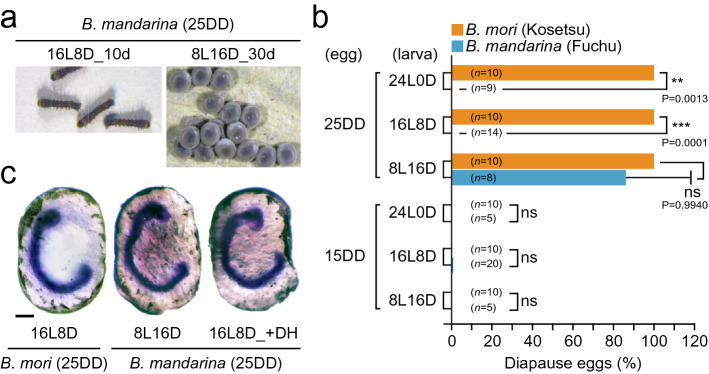
Temperature and photoperiodic diapause induction in *Bombyx mori* and *B. mandarina*. (**a**) The typical larvae and eggs of the wild mulberry silkworm *B. mandarina*. Eggs were incubated at 25DD; after that, larvae were reared at 16L8D or 8L16D. Progeny eggs hatched on 10 d after oviposition under the 16L8D condition, whereas progeny eggs never hatched by 30 d after oviposition under the 8L16D condition. (**b**) Effects of temperature and photoperiodic conditions during embryonic and larval stages on diapause egg-inducing activity in the Kosetsu strain of *B. mori* and a Fuchu population of *B. mandarina*. After eggs were incubated under the 25DD or 15DD condition, larvae were reared on 24L0, 16L8D, and 8L16D conditions. Each bar represents the mean ± SD of 5 − 20 animals. Significant differences represented in *B. mori* versus *B. mandarina* are in the same condition. ns, non-significant; **, *P* < 0.01; ***, *P* < 0.001. (**c**) Thionin staining of embryos 30 d after oviposition of domestic silkworm *B. mori* in 16L8D (25DD), and 30 d after oviposition of *B. mandarina* in both 8L16D and DH-injected silkworm in 16L8D (25DD). Scale bar = 200 µm.

We first confirmed the photoperiodic response in diapause induction of *B. mandarina* as described previously^27^. When eggs were incubated under 25DD during embryonic development, and thereafter, the larvae reared under long-day conditions, 16L8D and 24L0D, eclosed moth laid non-diapause eggs that hatched 10 d after oviposition (Fig. 1a,b). In contrast, most eggs were pigmented and never hatched 30 d after oviposition under short-day condition, 8L16D, for 25DD embryos. These pigmented eggs arrested normal embryogenesis, and the arrested stage was similar to that in diapause eggs in *B. mori* (Fig. 1a,c). The progeny eggs entered diapause under all photoperiodic conditions when embryos were reared under 25DD in *B. mori* (Fig. 1b), indicating that there were differences in the environmental cues also temperature or photoperiodic-dependent diapause induction in both strains. However, both strains never entered diapause for progeny from the 15DD condition (Fig. 1b).

### DH induces progeny diapause in *B. mandarina*

**Figure 2:**
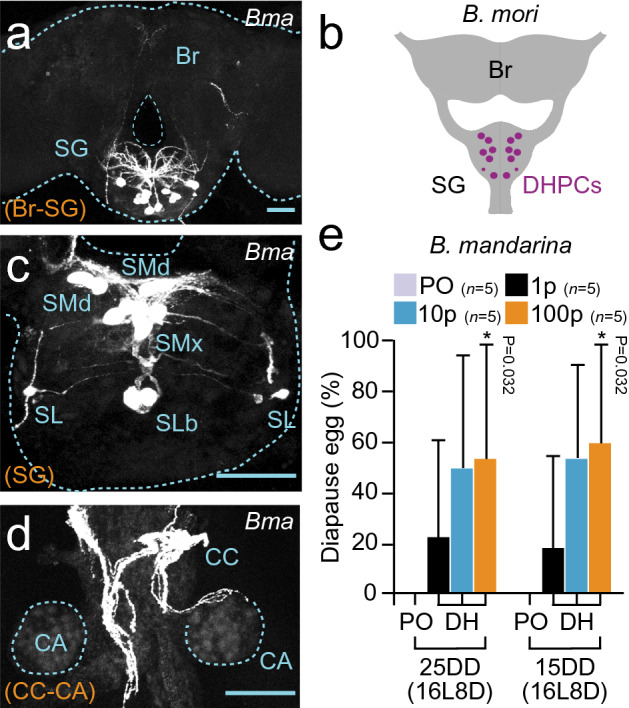
Diapause hormone (DH) induces egg diapause in *B. mandarina*. (**a**, **c**, **d**) Immunostaining of anti-DH[N] antibody in brain-subesophageal ganglion (SG) complex (**a**), SG (**c**), and corpus allatum (CA)** − **corpus cardiacum (CC) complex (**d**) of *B. mandarina* pupa Tissues were dissected out on 1 d after pupation from 8L16D (25DD) pupae. Scale bar = 100 µm. (**b**) Schematic drawing of pupal brain–SG complex in *Bombyx mori*. DH is produced in DH-PBAN-producing neurosecretory cells (DHPCs) located within the SG. (**e**) Diapause egg-inducing activity of DH injection. Peanut oil (PO) or each DH peptide concentration (1, 10, or 100 pmol) was injected into *B. mandarina* pupa reared under the 16L8D condition from 25 and 15DD embryos, and diapause egg-inducing activity was measured. Each bar represents the mean ± SD of five animals. Asterisks indicate significant differences versus PO injection. *, *P* < 0.05.

To clarify whether the DH signaling pathway is involved in diapause induction in *B. mandarina*, we determined the complementary DNA (cDNA) sequences of *DH-PBAN* and *DHR*. The *B. mandarina DH-PBAN* cDNA was cloned, and the deduced amino acid sequence of DH (BmaDH) was identical to that of *B. mori* DH (BmoDH)^28^ (Supplementary Fig. S1). We also found the homologous sequence to *B. mori DHR* (*BmoDHR*) cDNA using SilkBase (http://silkbase.ab.a.u-tokyo.ac.jp/cgi-bin/index.cgi). Using reverse transcription PCR (RT-PCR), we cloned *B. mandarina DHR* cDNA (*BmaDHR*; Acc. No. LC594680). The sequence of the deduced amino acids in BmaDHR represented a highly similar sequence in BmoDHR at 97%, including the seven transmembrane regions (TM1–TM7) (Supplementary Fig. S1). Next, immunostaining was performed using an anti-DH antibody. High-immunofluorescent signals were detected as a cluster of somata in SG (Fig. 2a,c), considering that these immunoreactive clusters of somata were identical to SMd, SMx, SLb, and SL neurons of DHPCs in *B. mori*^12^ (Fig. 2b,c). Furthermore, immunoreactive neurites with bead-like varicosities were detected in the CC and projected onto the corpus allatum (CA) surface to form blind ends like that of *B. mori* (Fig. 2d), suggesting that BmaDH was released to the hemolymph through these neurohemal organs.

Next, we injected synthetic DH into *B. mandarina* pupae developed from 25 and 15DD embryos and reared under the 16L8D condition (Fig. 2e). Although peanut oil-injected silkworms never oviposited diapause eggs, DH-injected silkworms oviposited diapause eggs in a dose-dependent manner. The diapause eggs were staged in the early embryonic stages, which was similar to the cases of diapause eggs of *B. mandarina* (8L16D) and *B. mori* (Fig. 1c, 16L8D_ + DH). Therefore, we concluded that the DH signaling pathway is conserved between *B. mori* and *B. mandarina* to induce progeny diapause.

### Cloning and functional analysis of TRPA1 orthologs in *B. mandarina*

**Figure 3:**
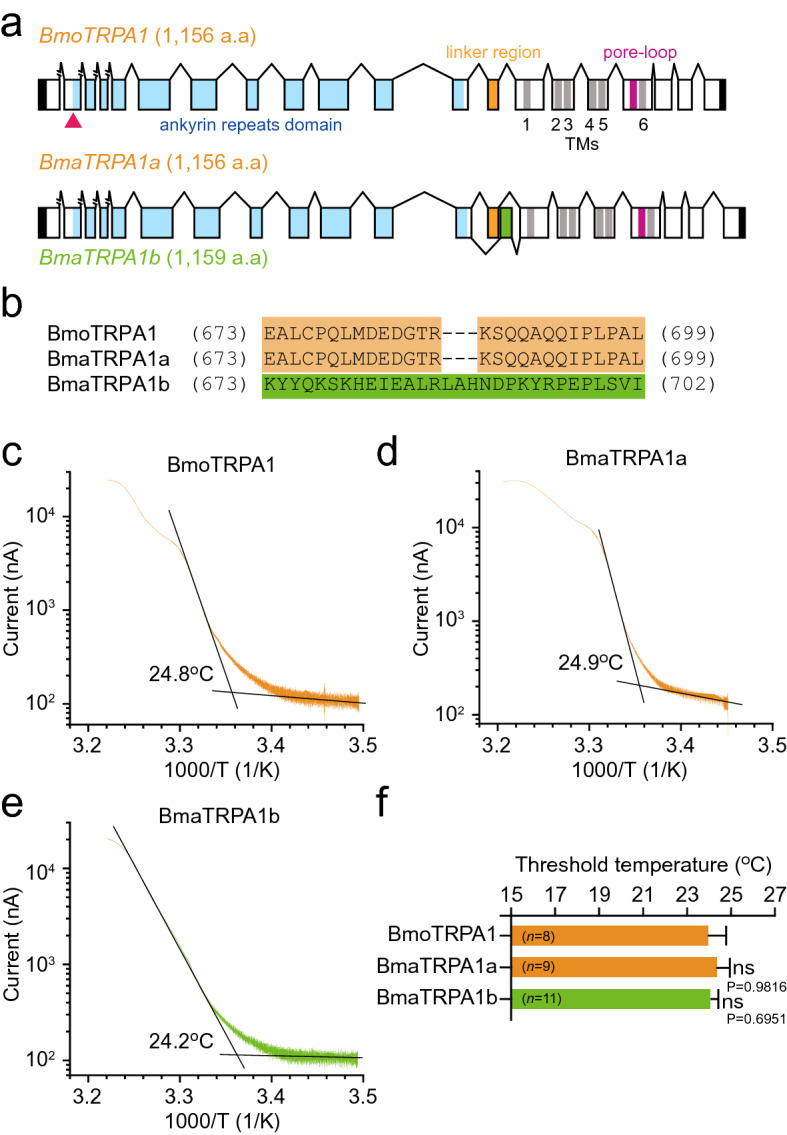
Cloning and functional analysis of *Bombyx mori* and *B. mandarina* TRPA1. (**a**) Schematic representations of the *B. mori TRPA1* (*BmoTRPA1*), *B. mandarina TRPA1a* (*BmaTRPA1a*), and *TRPA1b* (*BmaTRPA1b*). Boxes and lines represent exons and introns, respectively. Black, blue, orange, gray, and red boxes represent domains of untranslated regions, ankyrin repeat, linker region, transmembrane (1 ~ 6), and pore-loop, respectively. The red triangle indicates the TALEN target site. (**b**) Sequence alignment of the linker region between TRPA1s. (**c**–**e**) Arrhenius plots for the heat-induced BmoTRPA1, BmaTRPA1a, and BmaTRPA1b current shows a clear flex point in temperature dependency. Each temperature threshold for TRPA1 activation was defined as when the two linear-fitted lines crossed (i.e., a flex point). (**f**) Comparison of threshold temperature for each TRPA1. Each bar represents the mean ± SEM of 8–11 samples. Significant differences versus BmoTRPA1 were evaluated. ns, non-significant.

We cloned two TRPA1 cDNAs, namely *BmaTRPA1a* (Acc. No. LC597009.1) and *BmaTRPA1b* (Acc. No. LC597010.1), from *B. mandarina* eggs just before hatching. The amino acid sequences deduced from the two cDNAs (*BmaTRPA1a* and *BmaTRPA1b*) were highly similar to that of BmoTRPA1 at 99% and 97%, respectively (Fig. 3a and Supplementary Fig. S2). The *BmaTRPA1* genes are conserved in the functional domain consisting of ankyrin repeat domains (ANK1 ~ 17), transmembrane domains (TM1–6), and a pore-loop domain among the TRPA1 subfamily^29,30^. *BmaTRPA1a* and *BmaTRPA1b* transcripts were caused by alternative splicing of distinct exons encoding the linker region located between the last ankyrin repeat and the first transmembrane segment (Fig. 3a,b, and Supplementary Fig. S2). This structure resembles the alternative splicing variants of TRPA1 found in *Drosophila melanogaster* and *Aedes aegypti*^29,30^.

Next, channel properties of TRPA1 between the two species were compared by an electrophysiological approach. BmaTRPA1 or BmoTRPA1 were heterologously expressed in *Xenopus* oocytes, and ionic currents were observed. TRPA1 from both species was activated by heat. We investigated temperature sensitivity and determined the threshold temperature for activation for BmaTRPA1a and BmaTRPA1b as well as BmoTRPA1 with an Arrhenius plot (Fig. 3c–f). The average thermal activation threshold of BmoTRPA1 was 24.0 °C ± 0.8 °C (Fig. 3c). This value is slightly higher than that of BmoTRPA1 previously reported using mammalian cultured cells (approximately 21 °C)^14^, which might be caused by different cell types used for heterologous expression of TRPA1. The average thermal activation thresholds of BmaTRPA1a and BmaTRPA1b were 24.4 °C ± 0.6 °C and 24.0 °C ± 0.4 °C, respectively. No statistical difference was observed among thermal activation thresholds for BmoTRPA1, BmaTRPA1a, and BmaTRPA1b (Fig. 3f). Therefore, we concluded that the embryonic expression and temperature sensitivity of TRPA1 are conserved in both species.

### Knockout (KO) analysis of BmoTRPA1 in diapause induction

**Figure 4:**
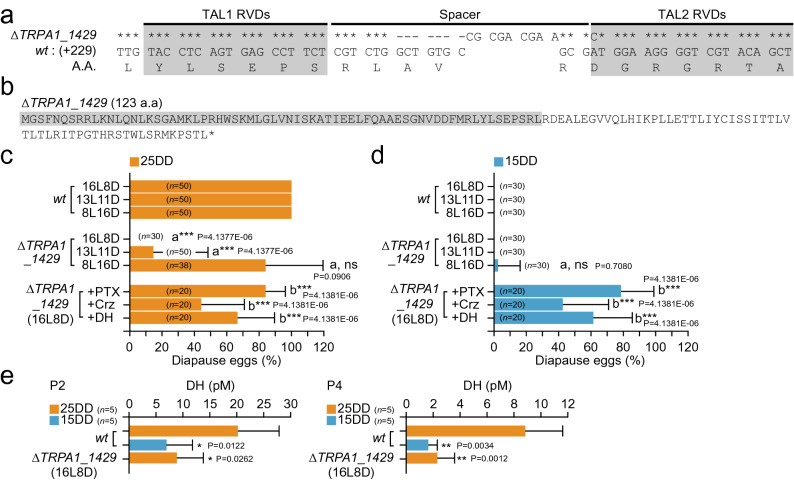
Knockout (KO) mutant of *BmoTRPA1*. (**a**) Gray boxes indicate the sequences of the TALEN target sites. The sequence of the *BmoTRPA1* gene of the mutant line *∆TRPA1_1429* that inserted, deleted, and replaced 17 bases between the spacer region and right TALEN binding site (TAL2 RVDs) are indicated. Asterisks indicate the bases identical to wild type (*wt*) sequences. (**b**) The *∆TRPA1_1429* gene encodes a truncated protein consisting of 123 amino acids. (**c**, **d**) Diapause egg-inducing activity in *∆TRPA1_1429*. The proportions of diapause eggs oviposited from *wt* and KO mutant female moths of 25DD (**c**) and 15DD (**d**) were measured as well as that of moths injected with plant alkaloid picrotoxin (PTX), corazonin (Crz), and diapause hormone (DH) at 50 µg, 1 nmol, and 100 pmol/pupa, respectively. Significant differences versus *wt* (a) or non-injected silkworm on 16L8D (b) were evaluated. (**e**) DH levels in hemolymph 2 (P2) and 4 (P4) d after pupation in both *wt* and *∆TRPA1_1429*. Significant differences versus *wt* (25DD) were evaluated. ns, non-significant; *, *P* < 0.05; **, *P* < 0.01; ***, *P* < 0.001.

Since both temperature-sensitive TRPA1 and DH signaling pathways are conserved in *B. mori* and *B. mandarina*, we hypothesized that there were differences in the downstream signal of TRPA1 activation to induce diapause between *B. mori* and *B. mandarina*. Hence, we constructed a KO mutant of *BmoTRPA1* to investigate the TRPA1-activated downstream pathway. We designed a transcription activator-like effector nuclease (TALEN) target in the second exon encoding the first ankyrin repeat (ANK1) (Figs. 3a, 4a, and Supplementary Fig. S2). We isolated a homozygous mutant in which both a 7-base sequence was deleted, and a 9-base sequence was inserted into the spacer region between two TALEN binding sites, and a single base pair was replaced in the right TALEN binding site (TAL2) and designated the mutant as *∆TRPA1_1429* (Fig. 4a,b). *∆TRPA1_1429* was considered a null mutant, which could not translate full-length *Bmo*TRPA1 due to a frameshift of *BmoTRPA1* cDNA. We then investigated whether the *∆TRPA1_1429* silkworm oviposited non-diapause eggs from 25 and 15DD (Fig. 4c,d). Although the wild type (*wt*) moths laid 100% of diapause eggs, all *∆TRPA1_1429* moths laid only non-diapause eggs when the larvae were reared under the long-day condition (16L8D). In this condition, the hemolymph DH levels of *∆TRPA1_1429* from 25DD embryos were extremely low compared with those of *wt* for both samples collected 2 and 4 days after pupation, resulting in decreased levels similar to the 15DD of *wt* (Fig. 4e). Furthermore, when picrotoxin (PTX), Crz, or DH were injected into *∆TRPA1_1429* pupa under the 16L8D condition, higher diapause egg-inducing activity was observed, suggesting that the GABAergic, Crz, and DH signaling pathways were retained in *∆TRPA1_1429* as those in 15DD silkworms (Fig. 4d).

However, the ratios of non-diapause eggs gradually decreased depending on the length of scotophase in *∆TRPA1_1429*. The percentages of diapause eggs were 14.7% and 84.6% in 13L11D and 8L16D, respectively (Fig. 4c). Taken together, we speculated that the BmoTRPA1-activated pathway is involved in diapause induction in the *wt* of Kosetsu through the GABAergic, Crz, and DH signaling pathways, because the disruption of the TRPA1-activated pathway in the BmoTRPA1 KO mutant could not operate the temperature-dependent diapause induction. Interestingly, BmoTRPA1 KO mutants’ progeny eggs exhibited photoperiodic diapause induction, as is the case with *B. mandarina*. This suggests that the *wt* of Kosetsu potentially possesses a signaling pathway involved in photoperiodic diapause induction that might be canceled by the strong linkage of the BmTRPA1-activated signal and DH signaling pathways. Consequently, the progeny diapause may be dependent on photo- or scotophase-length in *BmoTRPA1* KO mutants such as *B. mandarina*. To our knowledge, the temperature-dependent diapause induction did not occur in *B. mandarina*. Therefore, we propose that the temperature-dependent diapause induction phenotype was artificially selected and spread throughout *B. mori* populations during the ancient domestication process, given the fact that temperature regulation was much easier than photoperiodic control by humans. Moreover, both *B. mandarina* and the TRPA1 mutant of *B. mori* only entered diapause under short-day conditions at 25 °C but could not do so at 15 °C. Therefore, BmTRPA1 might play a partial role in signaling, and other unidentified additional factors (e.g., cold-sensitive thermosensors activated at ≤ 15 °C) may also be involved in diapause induction. Furthermore, as it is suggested that insect clocks are engaged in photoperiodic response, including diapause induction^31^, both the temperature and photoperiodic diapause induction may be mediated by common unidentified machinery such as circadian clocks. In this study, we investigated to elucidate the molecular mechanism of selectivity and integration of environmental cues concerning diapause induction. Our results demonstrated the usefulness of a comparative study using the Kosetsu strain of *B. mori* and a Japanese population of *B. mandarina* for further research on diapause induction.

## Methods

### Silkworms

For *B. mori*, the bivoltine Kosetsu strain was used in these experiments. Eggs were incubated under two different conditions: (1) at 25 °C under continuous darkness (25DD) to obtain diapause eggs in the *wt*, and (2) at 15 °C under continuous darkness (15DD) to obtain non-diapause eggs. Larvae were then reared on an artificial diet (Kuwano-hana for young silkworm, Gunma Artifical Diet Production Center, Takasaki, Gunma) at 25 °C–27 °C under 16-h light/8-h dark (16L8D), 13L11D, 8L16D, and 24L0D cycles at a relative humidity of 30–50%. Pupae used in the experiments were collected within 1 h after each ecdysis (referred to as day 0) to synchronize their subsequent development. Pupae were incubated at 25 °C to allow for adult development. The larvae of wild mulberry silkworm, *B. mandarina*, were collected from Tokyo University of Agriculture and Technology in Fuchu (35° 41′ N) and the Gunma Sericultural Technology Center at Maebashi (36° 24′ N). The oviposited non-diapause eggs were incubated at 25DD and 15DD. Larvae were reared on mulberry leaves at 25–27 °C under 16L8D, 8L16D, and 24L0D. Pupae used in the experiments were collected within 12 h after each ecdysis. Pupae were incubated at 25 °C to allow for adult development.

The percentage of diapause eggs was estimated by counting the number of eggs in diapause and those not in diapause in each egg batch after the non-diapause eggs hatched. The results are expressed as the average percentage of diapause in each egg batch^8,9^. For *B. mandarina*, the eggs were counted using a SZ61 stereo microscope (Olympus, Tokyo, Japan). Pupal-adult development lasted 12–26 d in these experiments, and a few pupae did not eclose within one month, as described in a previous report^32^. The uneclosed pupae were not used for calculation of the diapause eggs inducing activity.

### Thionin staining and immunostaining

Thionin staining was performed as previously described^8^. The eggs were fixed in Carnoy’s fixative for 1 d at 4 °C. The fixed eggs were treated with a gradient concentration of ethanol for 10 min. Following 5 min of boiling, the chorion was dissected, and the eggs were transferred into a thionine solution (0.07% thionine and 0.3% phenol dissolved in 80% ethanol) for 2 h. The stained eggs were rinsed with 80% ethanol four times and dehydrated with a gradient concentration of ethanol for 10 min after each rinse. The eggs were then soaked in benzene to make the yolk transparent. The embryos were observed using an M165 FC stereomicroscope (Leica, Wetzlar, Germany).

The immunoreaction procedures were adapted as previously reported^11^. The brain–SG complex or the CC and CA complex were dissected in fixative containing 4% paraformaldehyde, 7% picric acid, 10 mM MgCl_2_, 5 mM EGTA-NaOH, and 0.5 M HEPES–NaOH (pH 6.9) and incubated at 4 °C overnight. The fixed tissues were stored in 90% methanol containing 50 mM EGTA at − 20 °C until use. The fixed and stored tissues were hydrated through decreasing concentrations of methanol and washed with PBS containing 0.2% Tween-20 (PBT). Tissue samples were soaked in PBS containing 2% Tween-20 for two nights, washed with PBT, blocked with PBT containing 5% heat-inactivated goat serum (Wako, Osaka, Japan) and 2% BSA, and incubated with anti-DH[N]^11^ at 1:2500 at 4 °C overnight. The signal was detected with a Cy3-labeled IgG (Jackson ImmunoResearch Lab, West Grove, PA, USA) diluted to 1:1500 using an FV1000-D confocal microscope (Olympus).

### Injection of picrotoxin (PTX) and peptides

PTX was purchased from Sigma Aldrich (St. Louis, MI, USA). The chemical synthetic Crz peptide was purchased from Abbiotec (Escondido, CA, USA). DH was obtained from Operon Biotechnologies (Tokyo, Japan). PTX and peptides were dissolved in distilled water and peanut oil, respectively, and 10-µL solutions of various doses were injected into pupae a day after pupation through the intersegmental membrane between the second and third abdominal segments.

### cDNA cloning

Eggs were collected the day before hatching. Ovaries were dissected from pupae a day after pupation. Total RNA was extracted from eggs and ovaries using TRI-reagent (Molecular Research Center, Inc., Cincinnati, OH, USA) and then subjected to first-strand DNA synthesis using ReverTra Ace qPCR RT Master Mix with gDNA Removert (Toyobo, Osaka, Japan). By searching the highly-homologous sequences of each *DHR* and *TRPA1* cDNA for *B. mandarina* in the genomic database SilkBase (http://silkbase.ab.a.u-tokyo.ac.jp/cgi-bin/index.cgi), we designed the primers (Supplementary Table S1) to amplify each open reading frame sequence using RT-PCR.

### Electrophysiological assay

TRPA1 channel property was characterized by expressing the channel in *Xenopus laevis* oocytes, and ionic currents were measured by a two-electrode voltage clamp method as previously described^33,34^. In brief, *BmoTRPA1*, *BmaTRPA1a*, and *BmaTRPA1b* were cloned into a pOX plasmid vector, and complementary RNA (cRNA) was synthesized with the mMESSAGE mMACHINE SP6 kit (Thermo Fisher Scientific, Waltham, MA, USA) using a linearized pOX vector containing TRPA1 as a template. Fifty nanoliters of each TRPA1 cRNA (50 or 100 ng/μL) was then injected into defolliculated *Xenopus* oocytes, and ionic currents were observed 2–6 d post-injection. Oocytes were voltage-clamped at − 60 mV, and ionic currents were recorded using an OC-725C amplifier (Warner Instruments, Holliston, MA, USA) with a 1-kHz low-pass filter and digitized at 5 kHz using a Digidata 1440 Digitizer (Molecular Devices, San Jose, CA, USA). For thermal stimulation, heated or chilled ND96 bath solutions containing (mM) 96 NaCl, 2 KCl, 1.8 CaCl_2_, 1 MgCl_2_, and 5 HEPES (pH 7.6), were applied by perfusion. The temperature was monitored with a thermistor located just beside the oocytes using a TC-344B controller (Warner Instruments). Heat-evoked currents for TRPA1 were obtained from *Xenopus* oocytes from two independent preparations, and apparent thermal activation thresholds for TRPA1 were determined with an Arrhenius plot that was generated using Clampfit 10.4 (Molecular Devices) and Origin 9 J (OriginLab, Northampton, MA, USA).

### TALEN construction and screening of KO silkworm

The TALEN-based mutant lines were constructed according to the previous reports^8,35^. Briefly, TALEN targets were searched using TAL Effector Nucleotide Targeter 2.0 (https://tale-nt.cac.cornell.edu) in the coding regions of target genes. DNA constructs containing TAL segments were prepared using a Golden Gate TALEN kit (Addgene, Cambridge, MA, USA). TALEN mRNAs were then synthesized using the mMESSAGE mMACHINE T7 Ultra kit (Ambion, Carlsbad, CA, USA). The mRNA of each TALEN was mixed at a concentration of 0.5 µg/µL for microinjection. Non-diapause eggs of the Kosetsu strain were collected within 1 h after oviposition during the syncytial blastoderm stage. The TALEN mRNA mixture was injected into the eggs using a glass needle (uMPm-02; Daiwa Union, Iida, Japan) attached to a manipulator (kaikopuchu-STDU1; Daiwa Union) and FemtoJet (Eppendorf, Hamburg, Germany).

For screening germline mutagenesis, G_0_ adults were mated with *wt*. The oviposited G_1_ eggs were collected, and approximately ten eggs from each brood were pooled for genomic DNA extraction using DNAzol reagent (Thermo Fisher Scientific). The DNA fragment containing the targeted region of interest was amplified by PCR using Takara Ex Taq (Takara, Tokyo, Japan) and specific primers (Supplementary Table S1). To test for mutagenesis, the PCR product of *BmoTRPA1* was treated with the Surveyor Mutation Detection kit (IDT, Tokyo, Japan). The mutated PCR products were checked by sequencing. The broods containing mutated sequences were reared, and mutated G_1_ adults were crossed with the siblings carrying the same mutation. Homozygous mutants were obtained after confirmation by sequencing of the target region in the G_2_ or G_3_ egg genome.

### DH extraction and titer measurement

DH was extracted from the hemolymph as previously described^8^. DH extraction was performed at ZT6 (ZT = zeitgeber time, ZT = 0 corresponds to light on). DH levels were measured using a time-resolved fluoroimmunoassay as described previously^8^.

### Statistical analysis

Statistical parameters, including definitions and exact values of *n*, are provided in the relevant figures or corresponding figure legends. Statistical analyses were performed in Microsoft Excel 2011 with the software add-in Toukei-Kaiseki Ver. 3.0 (Esumi, Tokyo, Japan). The significance of differences in diapause egg-inducing activity was evaluated using the Steel–Dwass test. Other data were compared using Student’s *t*-tests. Data are expressed as the mean ± SD. *P* < 0.05 was considered significant; ns, non-significant; *, *P* < 0.05; **, *P* < 0.01; ***, *P* < 0.001.

## Supplementary Information

Supplementary Information.

## Data Availability

The datasets in the current study are available from the corresponding author on reasonable request.
