# Supplementary material for: Comparisons in temperature and photoperiodic-dependent diapause induction between domestic and wild mulberry silkworms
Source: Sci Rep. 2021 Apr 13;11:8052. doi: 10.1038/s41598-021-87590-4 (PMC8044193; doi:10.1038/s41598-021-87590-4)
Supplement: Supplementary file 1 — Supplementary Information. [file 41598_2021_87590_MOESM1_ESM.pdf]

# Supplementary Table S1

List of primers*

| | |
|---|---|
| a. For cDNA cloning | |
| 1. DHR | F: 5′-CGTCACGTAACGTCAACATTCGCGGTACGC-3′ |
| | R: 5′-TAATTACTTACTAAAACGGAATGTATTTG-3′ |
| 2. TRPA1 | F: 5′-GTCATGACATTTTGTGGAACAACGGGCCTA-3′ |
| | R: 5′-TCAAGTCGACTTGTTAAATGATGCAGCTCG-3′ |
| b. For screening of the Δ*TRPA1* mutant | |
| | F: 5′-AGTTTTTTTGTACAGGCTGCTGAAAGCGGA-3′ |
| | R: 5′-TCTCGGTTTGCATCGCCAATAAGC-3′ |

*F; Forward primer, R; Reverse primer.

a

```
BmoDH;   TDMKDESDRGAHSERGALWFGPRL  24
BmaDH;   TDMKDESDRGAHSERGALWFGPRL  24
         ************************
```

b

```
                                             TM1
BmoDHR;  MNS--ETINDTANASRPVDSTRVFGPQRDTLYIVLPITIIYTFIFVSGLLGNIFTCIVIV  58
BmaDHR;  MNSLNETLNDTANASRPVDSTRVFGPQRDTLYIVLPITIIYTFIFISGLLGNIFTCIVIV  60
         ***  ** ************************************* **************
             TM2                                       TM3
BmoDHR;  RNKNLHTATNYYLFSLAISDLLLLVSGMPQEMYSIWSKWPYVFGHTFCVIRGLAAETSTN 118
BmaDHR;  RNKNLHTATNYYLFSLAISDLLLLVSGMPQEMYSIWSKWPYVFGHTFCVIRGLAAETSTN 120
         ************************************************************
                                             TM4
BmoDHR;  ASVLTITLFTIERYLAICHPFVSHKMSKLSRAVKHVVLLWVAALALALPQALQFGIRQYQ 178
BmaDHR;  ASVLTITLFTIERYLAICHPFVSHKMSKLSRAVKHVVLLWVAALALALPQALQFGIRQYQ 180
         ************************************************************
                          TM5
BmoDHR;  GVIMCLQTRVIIEHSFEISTFLFFLAPMVLITVLYSFIGLKLREKSNVKEQNQNDFESSI 238
BmaDHR;  GVIMCLQTRVIIEHSFEISTFVFFLAPMVLITVLYSFIGLKLREKSNVKEQNQNDFESSI 240
         ********************* **************************************
                             TM6
BmoDHR;  RYSHKMCRKPSQSTRRVIKMLVAVVVAFFICWAPFHAQRLVAIYGTNENHLAKSPILFSV 298
BmaDHR;  RYSHKMCRKPSQSTRRVIKMLVAVVVAFFVCWAPFHAQRLVAIYGTNENHLAKSPILFSV 300
         ***************************** ******************************
           TM7
BmoDHR;  YLFLTYISGIFYYMSTCINPILYHIMSNKFRDAFKMTLCCCGTRNDTAVKRSSYTAMAFV 358
BmaDHR;  YLFLTYISGICYYMSTCINPILYHIMSNKFRDAFKMTLCSCGTRNDTAVKRSSYTAMAFV 360
         ********** **************************** ********************

BmoDHR;  RHPTSSGTSNSGNSIRNETNLQSKTRRTNGRDKILNDAHVCRNGTLSSAAVGKPDSRTNG 418
BmaDHR;  RNPTSSGTSNSGNSIRNETNLQSKTRRTNSRDKILNDAHVCGNGTLSSAAVGKPDSRTNG 420
         * *************************** *********** ******************

BmoDHR;  DRPLDRNLINETYFNTNC 436
BmaDHR;  DRPLDRNLINETYFNTNC 438
         ******************
```

**Supplementary Figure S1.** Alignments of amino acid sequences of diapause hormone (DH) and DH receptor (DHR). The deduced amino acid sequences of *DH-PBAN* (**a**) and *DHR* (Acc. No. LC594680) (**b**) cDNAs in *Bombyx mori* (Bmo) and *B. mandarina* (Bma) were aligned. Identical amino acids are indicated by asterisks. The putative transmembrane domains (TM1~TM7) are indicated.

```
BmoTRPA1;   MGSFNQSRRLKNLQNLKSGAMKLPRHWSKMLGLVNISKATIEELFQAAESGNVDDFMRLY 60
BmaTRPA1a;  MGSFNQSRRLKNLQNLKSGAMKLPRHWSKMLGLVNISKATIEELFQAAESGNVDDFMRLY 60
BmaTRPA1b;  MGSFNQSRRLKNLQNLKSGAMKLPRHWSKMLGLVNISKATIEELFQAAESGNVDDFMRLY 60
            ************************************************************
            TAL RVDs   ANK1                               ANK2
BmoTRPA1;   LSEPSRLAVRDGRGRTAAHQASARNNTNILHFINNYAGDLNAKDNAGNTPLHVAVENEAL 120
BmaTRPA1a;  LSEPSRLAVRDGRGRTAAHQASARNNTNILHFINNYAGDLNAKDNAGNTPLHVAVENEAL 120
BmaTRPA1b;  LSEPSRLAVRDGRGRTAAHQASARNNTNILHFINNYAGDLNAKDNAGNTPLHVAVENEAL 120
            ************************************************************
                             ANK3                                 ANK4
BmoTRPA1;   DAIEYLLQQHVETSVLNEKCQAPIHMATELNKVSVLQVFAKYKSLFNVNQGGEHGRTALH 180
BmaTRPA1a;  DAIEYLLQQHVETSVLNEKCQAPIHMATELNKVSVLQVFAKYKSLFNVNQGGEHGRTALH 180
BmaTRPA1b;  DAIEYLLQQHVETSVLNEKCQAPIHMATELNKVSVLQVFAKYKSLFNVNQGGEHGRTALH 180
            ************************************************************
                                          ANK5
BmoTRPA1;   FAAIHDHDLCAKILITDLDAEWKKPCNNGYYPIHEAAKNASSRTIEVFLQWGEQRGCTRE 240
BmaTRPA1a;  FAAIHDHDLCAKILITDLDAEWKKPCNNGYYPIHEAAKNASSRTIEVFLQWGEQRGCTRE 240
BmaTRPA1b;  FAAIHDHDLCAKILITDLDAEWKKPCNNGYYPIHEAAKNASSRTIEVFLQWGEQRGCTRE 240
            ************************************************************
                    ANK6                                ANK7
BmoTRPA1;   QMISLYDNEGNVPLHSAVHGGDIKAVELCLRSGAKISTQQHDLSTPVHLACAQGALDIVK 300
BmaTRPA1a;  QMISLYDNEGNVPLHSAVHGGDIKAVELCLRSGAKISTQQHDLSTPVHLACAQGALDIVK 300
BmaTRPA1b;  QMISLYDNEGNVPLHSAVHGGDIKAVELCLRSGAKISTQQHDLSTPVHLACAQGALDIVK 300
            ************************************************************
                             ANK8                              ANK9
BmoTRPA1;   LMFTMQPKEKHACLTSCDVQKMTPIHCAAMFDLPEIVNYLINEGSDINPLDKERRSPLLL 360
BmaTRPA1a;  LMFTMQPKEKHACLTSCDVQKMTPIHCAAMFDHPEIVNYLINEGSDINPLDKERRSPLLL 360
BmaTRPA1b;  LMFTMQPKEKHACLTSCDVQKMTPIHCAAMFDHPEIVNYLINEGSDINPLDKERRSPLLL 360
            ******************************** ***************************
                                    ANK10
BmoTRPA1;   AASRAGWRTVHTLIRLGADIQLKDINSRNVLHLVVMNGGRLEDFAASCKDHCEKSLLQLL 420
BmaTRPA1a;  AASRAGWRTVHTLIRLGADIQLKDINSRNVLHLVVMNGGRLEDFAASCKDHCEKSLLQLL 420
BmaTRPA1b;  AASRAGWRTVHTLIRLGADIQLKDINSRNVLHLVVMNGGRLEDFAASCKDHCEKSLLQLL 420
            ************************************************************
                ANK11                                ANK12
BmoTRPA1;   NEKDNTGCSPLHYASREGHIRSLENLIRLGACINLKNNNNESPLHFAARYGRFHTACQLL 480
BmaTRPA1a;  NEKDNTGCSPLHYASREGHIRSLENLIRLGACINLKNNNNESPLHFAARYGRFHTACQLL 480
BmaTRPA1b;  NEKDNTGCSPLHYASREGHIRSLENLIRLGACINLKNNNNESPLHFAARYGRFHTACQLL 480
            ************************************************************
                          ANK13                               ANK14
BmoTRPA1;   DSDKGTFIINESDGEGLTPLHIASREGHTRVVHLLLNRGALLHRDHNGRNPLHLAAMSGY 540
BmaTRPA1a;  DSDKGTFIINESDGEGLTPLHIASREGHTRVVHLLLNRGALLHRDHNGRNPLHLAAMSGY 540
BmaTRPA1b;  DSDKGTFIINESDGEGLTPLHIASREGHTRVVHLLLNRGALLHRDHNGRNPLHLAAMSGY 540
            ************************************************************
                                ANK15                            ANK16
BmoTRPA1;   TKTIELLHSVHSHLLDQIDKDGNTSLHLATMENKPSSIALLLSMGCRLSYNNMDMSAIDY 600
BmaTRPA1a;  TKTIELLHSVHSHLLDQIDKDGNTSLHLATMENKPSSIALLLSMGCRLSYNNMDMSAIDY 600
BmaTRPA1b;  TKTIELLHSVHSHLLDQIDKDGNTSLHLATMENKPSSIALLLSMGCRLSYNNMDMSAIDY 600
            ************************************************************
                                       ANK17
BmoTRPA1;   AIYYKFPEAALAMVTHEQRAKEVMALRSDRHPCVTLALIAYMPRVFEAVQDKCITKANCK 660
BmaTRPA1a;  AIYYKFPEAALAMVTHEQRAKEVMALRSDRHPCVTLALIAYMPRVFEAVQDKCITKANCK 660
BmaTRPA1b;  AIYYKFPEAALAMVTHEQRAKEVMALRSDRHPCVTLALIAYMPRVFEAVQDKCITKANCK 660
            ************************************************************
                  linker region
BmoTRPA1;   KDSKSFYIKYSFEALCPQLMDEDGTR---KSQQAQQIPLPALNAMVAHGRVELLAHPLSQ 717
BmaTRPA1a;  KDSKSFYIKYSFEALCPQLMDEDGTR---KSQQAQQIPLPALNAMVAHGRVELLAHPLSQ 717
BmaTRPA1b;  KDSKSFYIKYSFKYYQKSKHEIEALRLAHNDPKYRPEPLSVINAMVAHGRVELLAHPLSQ 720
            ************                         **   ******************
                  TM1
BmoTRPA1;   KYLRMKWNSYGKYFHLVNVLFYCIFLIFVTVYSYLLMEHVNPINKRERSRVGDVYYNYTA 777
BmaTRPA1a;  KYLQMKWNSYGKYFHLVNVLFYCIFLIFVTVYSYLLMEHVNPINKRERSRVGDVYYNYTA 777
BmaTRPA1b;  KYLQMKWNSYGKYFHLVNVLFYCIFLIFVTVYSYLLMEHVNPINKRERSRVGDVYYNYTA 780
            *** ********************************************************
                           TM2                        TM3
BmoTRPA1;   TNRTQTNWDIDADFEAILIMYTSSVVIMVYISVCMMREAYNLKQQKWHYIVDPSNLVSWT 837
BmaTRPA1a;  TNRTQTNWDIDADFEAILIMYTSSVVIMVYISVCMIREAYNLKQQKWHYIVDPSNLVSWT 837
BmaTRPA1b;  TNRTQTNWDIDADFEAILIMYTSSVVIMVYISVCMIREAYNLKQQKWHYIVDPSNLVSWT 840
            *********************************** ************************
                           TM4
BmoTRPA1;   LYISATITVFPTLYGHYSNYQFSAASITVFLSWFELLLLLQRFDQVGIYVVMFLEILQTL 897
BmaTRPA1a;  LYISATITVFPTLYGHYSNYQFSAASITVFLSWFELLLLLQRFDQVGIYVVMFLEILQTL 897
BmaTRPA1b;  LYISATITVFPTLYGHYSNYQFSAASITVFLSWFELLLLLQRFDQVGIYVVMFLEILQTL 900
            ************************************************************
            TM5                                 pore-loop
BmoTRPA1;   IKVLMVFSILIIAFGLAFYILLSKGNHLSFSNIPIALMRTFAMMLGELDFVGTYVQPYYK 957
BmaTRPA1a;  IKVLMVFSILIIAFGLAFYILLSKGNHLSFSNIPIALMRTFAMMLGELDFVGTYVQPYYK 957
BmaTRPA1b;  IKVLMVFSILIIAFGLAFYILLSKGNHLSFSNIPIALMRTFAMMLGELDFVGTYVQPYYK 960
            ************************************************************
                     TM6
BmoTRPA1;   DDSDIILPFPIPTFIILALFMILMPILLMNLLIGLAVGDIESVRRNAQLKRLAMQVVLHT 1017
BmaTRPA1a;  DDSDIILPFPIPTFIILALFMILMPILLMNLLIGLAVGDIESVRRNAQLKRLAMQVVLHT 1017
BmaTRPA1b;  DDSDIILPFPIPTFIILALFMILMPILLMNLLIGLAVGDIESVRRNAQLKRLAMQVVLHT 1020
            ************************************************************

BmoTRPA1;   ELERKLPAFLLEKVDKLELIEYPNNKKCKLGFLDLILHKWFCNPFTEDTGLDLVLENNDD 1077
BmaTRPA1a;  ELERKLPAFLLEKVDKLELIEYPNNKKCKLGFLDLILHKWFCNPFTEDTGLDLVLENNDD 1077
BmaTRPA1b;  ELERKLPAFLLEKVDKLELIEYPNNKKCKLGFLDLILHKWFCNPFTEDTGLDLVLENNDD 1080
            ************************************************************

BmoTRPA1;   YVTEQMEKQKRKFREMQNVLDQQYLLLRLIVQKMEIKTEADDVDEGVSPNDTKVVPRWSS 1137
BmaTRPA1a;  YVTEQMEKQKRKFREMQNVLDQQYLLLRLIVQKMEIKTEADDVDEGVSPNDTKVVPRWSS 1137
BmaTRPA1b;  YVTEQMEKQKRKFREMQNVLDQQYLLLRLIVQKMEIKTEADDVDEGVSPNDTKVVPRWSS 1140
            ************************************************************

BmoTRPA1;   HRNRKKLHSARAASFNKST 1156
BmaTRPA1a;  HRNRKKLHSARAASFNKST 1156
BmaTRPA1b;  HRNRKKLHSARAASFNKST 1159
            *******************
```

**Supplementary Figure S2.** Alignment of amino acid sequences of *Bombyx mori* and *B. mandarina* TRPA1. The deduced amino acid sequences of the *TRPA1* cDNA (Acc. Nos. AB703646.1, LC597009.1, and LC597010.1) in *B. mori* (Bmo) and *B. mandarina* (Bma) were aligned. Identical amino acids are indicated by asterisks. The TAL repeat variable diresidues (RVDs), ankyrin repeat (1~17), linker region, transmembrane (1~6), and pore-loop domains are indicated.
